# Response of pedogenic magnetite to changing vegetation in soils developed under uniform climate, topography, and parent material

**DOI:** 10.1038/s41598-017-17722-2

**Published:** 2017-12-14

**Authors:** Daniel P. Maxbauer, Joshua M. Feinberg, David L. Fox, Edward A. Nater

**Affiliations:** 1 Institute for Rock Magnetism, University of Minnesota, Minneapolis, MN 55455 USA; 2 Department of Earth Sciences, University of Minnesota, Minneapolis, MN 55455 USA; 3 Department of Soil, Water, and Climate, University of Minnesota, Saint Paul, MN 55108 USA; 4 Present Address: Geology Department, Carleton College, Northfield, MN 55057 USA

## Abstract

Pedogenesis produces fine-grained magnetic minerals that record important information about the ambient climatic conditions present during soil formation. Yet, differentiating the compounding effects of non-climate soil forming factors is a nontrivial challenge that must be overcome to establish soil magnetism as a trusted paleoenvironmental tool. Here, we isolate the influence of vegetation by investigating magnetic properties of soils developing under uniform climate, topography, and parent material but changing vegetation along the forest-prairie ecotone in NW Minnesota. Greater absolute magnetic enhancement in prairie soils is related to some combination of increased production of pedogenic magnetite in prairie soils, increased deposition of detrital magnetite in prairies from eolian processes, or increased dissolution of fine-grained magnetite in forest soils due to increased soil moisture and lower pH. Yet, grain-size specific magnetic properties associated with pedogenesis, for example relative frequency dependence of susceptibility and the ratio of anhysteretic to isothermal remanent magnetization, are insensitive to changing vegetation. Further, quantitative unmixing methods support a fraction of fine-grained pedogenic magnetite that is highly consistent. Together, our findings support climate as a primary control on magnetite production in soils, while demonstrating how careful decomposition of bulk magnetic properties is necessary for proper interpretation of environmental magnetic data.

## Introduction

Magnetic properties of soils are an important archive of climatic and environmental conditions present during soil formation^1–11^. Fine grained superparamagnetic (SP; <30 nm) and stable single domain (SSD; 30–75 nm) magnetite is produced by bacterially induced redox processes associated with wet and dry cycling in well-drained soils^3,8,12^. Soil formed magnetite is often exposed to oxic conditions that promote partial maghemitization during dry periods^13,14^. This population of SP/SSD magnetite and partially-oxidized magnetite, referred to together as pedogenic magnetite, is integrated into the pre-existing population of detrital magnetic minerals, often of coarser multidomain (MD; 100–300 nm and larger) or pseudo-single domain (PSD; in between SD and MD) grain sizes, that are derived from the physical weathering of parent material and/or deposited by eolian processes. Over time, mixtures of both pedogenic and detrital magnetic minerals are subjected to a range of continuing pedogenic processes that vary in response to factors such as climate, vegetation, topography, time, and parent material^15^, and act to produce, transform, or destroy magnetic minerals^10^. As a result, the magnetic minerals in soils record a kind of running average of local environmental conditions that extends over hundreds to thousands of years. Researchers interested in interpreting the ambient climate conditions during soil formation are challenged to disentangle mixed magnetic mineral assemblages in order to relate magnetic properties of soils with climate^10,11,16,17^.

Many of the more influential previous studies on soil magnetism sought to establish empirical relationships between magnetic properties and specific climate parameters, such as mean annual precipitation (MAP) or mean annual temperature (MAT)^1,2,6,9,18,19^. Magnetic paleoclimate proxies have proven to be powerful tools for reconstructing climate variability, particularly on the Chinese Loess Plateau^10^. However, regional differences and large uncertainties associated with magnetic proxies currently limit their applicability in other systems and in deep-time^11,20,21^. Pedogenic magnetite production in soils is mechanistically described as a function of the soil moisture balance (W), which is defined as the ratio between MAP and the potential evapotranspiration (PET) for a given environment^8^. PET is dependent on a variety of factors including climate and vegetation^8^. Targeted studies investigating the influence of other soil factors have improved our understanding into how the duration of soil development^22–25^, parent material^26–28^, and topography^29,30^ impact the magnetic mineralogy of soils. Yet, the role of vegetation on magnetic mineral assemblages in soils remains largely unconstrained.

**Figure 1:**
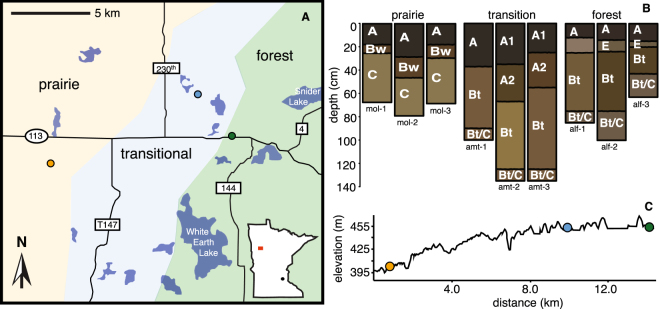
Site details and soil profiles. (**A**) Map of soil sampling localities within Minnesota (inset shown in bottom right of panel A, sampling locality is highlighted by the orange square, the black dot indicates Twin Cities area). Sampling localities within forest (dark green), prairie (orange), and transitional (blue) zones indicated with colored symbols. Map redrawn in Adobe Illustrator from Google Earth imagery (Map data: Google, DigitalGlobe). (**B**) Soil profiles and horizon designations for A, E, B, and C horizons. Labels below profiles correspond to specimen labels included in the Supplemental File. (**C**) Elevation profile for sampling sites along transect.

Here, we investigate magnetic properties of soils forming across the forest-to-prairie transition in NW Minnesota to evaluate the influence of changing vegetation and soil type on populations of pedogenic and detrital magnetic minerals (Fig. 1 and Figs S1, S2, and S3). Soils along the study transect have developed on Des Moines Lobe glacial till capped by a thin layer of loess since the last glacial retreat^31,32^. Despite strong E-W gradients in climate across the broader forest-prairie boundary in Minnesota, climate across our more restricted study transect is highly uniform (MAT = 4.6 °C, MAP = 650 mm yr^−1^, see seasonal distribution in Fig. S4; data from the PRISM Climate Group, Oregon State University, http://prism.oregonstate.edu, 9 March 2016) and all soils were sampled from stable uplands on relatively subtle topography (see Fig. 1) that has been mostly undisturbed (see Figs S1, S2, and S3 and discussion below). Vegetation differences along the transect are controlled by episodic natural fires, which act to reestablish prairie post-burning^31,33^. Given changes in vegetation across our study transect, we expect variations in W to be related to vegetation changes, not climate. Increased PET in the prairie will act to decrease W and may lead to an increase in the production of pedogenic magnetite in prairie soils^8^. If pedogenic production of magnetic minerals is controlled by climate alone, we expect to observe consistency in magnetic properties that isolate only the pedogenic population of magnetic minerals. Conversely, variability in magnetic properties can be taken as an indication that soil processes governed by vegetation changes and soil moisture balance are controlling magnetic mineral formation and/or dissolution.

## Results

**Figure 2:**
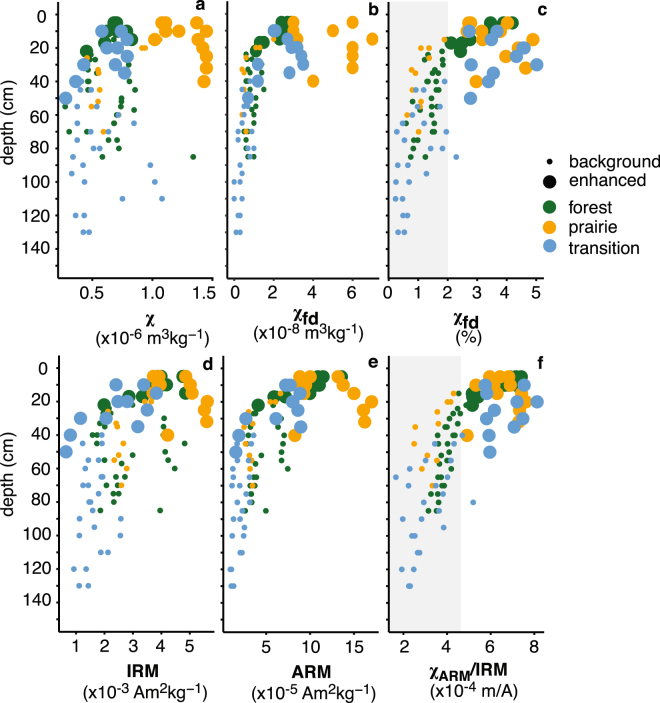
Magnetic properties with depth for soils across study transect (sample location indicated by color). The data reported here are magnetic susceptibility (*χ*); (**a**) absolute frequency dependence (*χ*_*fd*_) expressed as the difference between high (4650 Hz) and low (465 Hz) frequency susceptibility measurements; (**b**) relative frequency dependence of susceptibility (*χ*_*fd*_) expressed as a % (**c**) isothermal remanent magnetization (*IRM)* (**d**) anhysteretic remanent magnetization (*ARM*) (**e**) and the ratio of the susceptibility of *ARM* (*χ*_*ARM*_) to *IRM*. (**f**) Background (small symbols) and enhanced (large symbols) specimen in all cases are determined by criteria highlighted by shaded boxes in panels c and f, where enhanced specimen where greater than thresholds for *χ*_*fd*_ and *χ*_*ARM*_/*IRM* in both cases.

**Figure 3:**
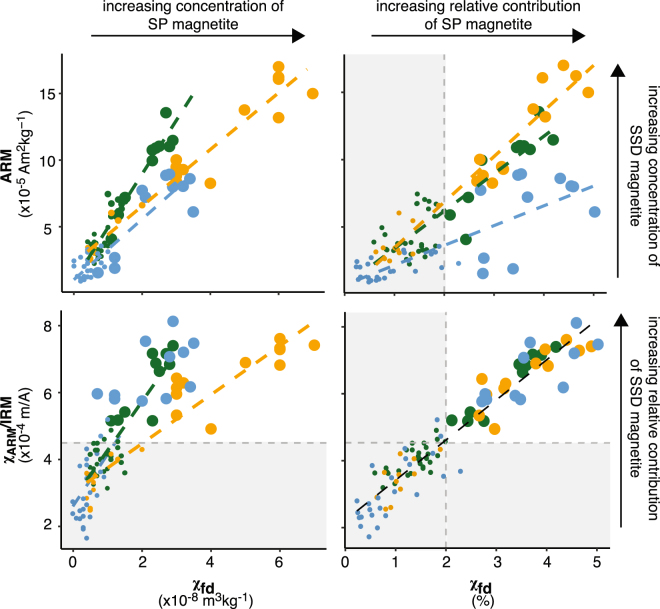
Cross plots of concentration dependent and independent magnetic properties that are sensitive to grain size variations in magnetite. Arrows and annotations indicate general trends in how each parameter is interpreted. Relative contribution refers to how much a particular grain size fraction of magnetite contributes to the bulk magnetic property of a given sample. Full descriptions and interpretation of these cross plots are provided in the text.

Concentration dependent magnetic properties in the upper 50 cm change markedly across the prairie-forest transect. For example, magnetic susceptibility (*χ*, Fig. 2a) ranges between 4 × 10^−7^ and 15 × 10^−7^ m^3^kg^−1^ and is significantly greater in enhanced prairie specimens compared with enhanced specimens in the forest and transitional soils (p < 0.001 for unpaired t-tests and Wilcoxon Signed Rank tests; see Methods and Fig. 2 for enhancement criteria). Saturation magnetization *M*_*s*_, Fig. S5) largely mirrors magnetic susceptibility within profiles. Both *χ* and *M*_*s*_ are induced magnetizations (measured in the presence of a magnetic field) and include contributions from magnetic minerals of all grain sizes. A frequent measure of the abundance of SP magnetite is the frequency dependence of susceptibility^34^ (*χ*_*fd*_ in units of m^3^kg^−1^ or %). The absolute frequency dependence (the difference between low and high frequency susceptibility; Figs 2b and 3; see Methods) increases with increasing concentration of SP magnetite^34^. Here, absolute frequency dependence increases in all topsoil across the transect, but is considerably greater in enhanced prairie topsoils relative to forest and transitional soils. Low-field isothermal and anhysteretic remanent magnetizations (*IRM* and *ARM*, respectively; see Fig. 2d,e) are more consistent between sampling biomes. Remanent magentizations are measured in the abscense of any magnetic field, and notably do not include any contributions from SP grains^35^.

Grain size dependent magnetic properties, when normalized to concentration, are comparatively more homogeneous across the vegetation transect. These magnetic parameters increase as the contribution of a particular grain size fraction to the samples total magnetization increases. For instance, the relative frequency dependence of susceptibility (*χ*_*fd*_ as a percentage of *χ*, Figs 2c and 3) and the ratio of the susceptibility of *ARM* to *IRM* (*χ*_*ARM*_/*IRM*, Fig. 2f) are relative indicators for the abundance of SP and SSD magnetite, respectively. Both properties are generally equivalent across the transect (p > 0.05 for all unpaired t-tests and Wilcoxon Signed Rank tests). Relative *χ*_*fd*_ and *χ*_*ARM*_/*IRM* are both increased in the upper soil horizons for all profiles and display trends consistent with a classical magnetically enhanced soil profile^3,8,11^. Cross plots of concentration-dependent and normalized grain-size sensitive magnetic properties are displayed in Fig. 3 and highlight the relationship between SP and SSD magnetite across the study transect. Full results for all parameters are available in Supplementary Fig. S5 and the data spreadsheet is available online.

**Figure 4:**
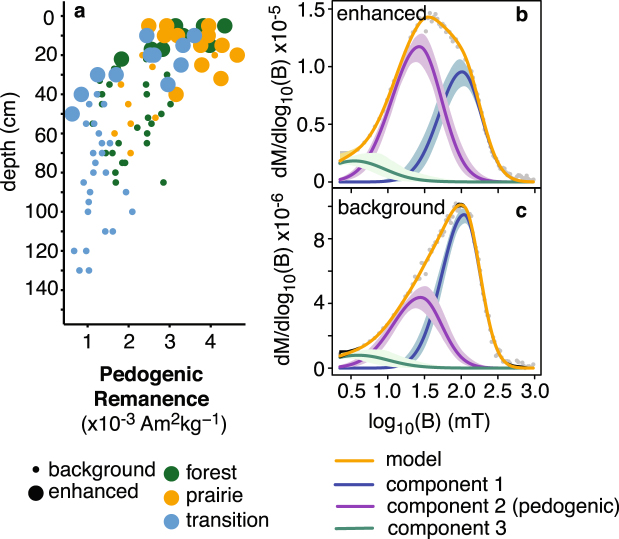
Results of coercivity analysis showing pedogenic remanence (**a**) and an example fit for enhanced (**b)** (M-2-01) and background, (**c)** (M-2-09) specimen. Pedogenic remanence is calculated based on the proportion of remanence at 1 T (*M*_*r*_) held by component 2 within the mixing model^36^. Parameters describing component 2 are highly consistent with observations of pedogenic magnetite from previous work^6,11,40^.

Unmixing coercivity distributions derived from backfield remanence curves resulted in a three component model fit for all specimens. Each component is described by its characteristic median coercive field (*B*_*h*_) and dispersion parameter (*DP*; one standard deviation in log10 space)^11^. Example fit results are shown in Fig. 4. Component parameters were consistent across the transect and did not show systematic variations with changes in vegetation and soil type. A high coercivity component (HCC, component 1) is characterized by a *B*_*h*_ of 1.97 ± 0.02 log10 mT (93.7 mT) and a *DP* of 0.29 ± 0.02. Mean *B*_*h*_ for an intermediate covercivity component (ICC, component 2) is 1.38 ± 0.03 log10 mT (24.0 mT) with a *DP* of 0.35 ± 0.02. Lastly, a low-coercivity component (LCC, component 3) has mean *B*_*h*_ of 0.53 ± 0.10 log10 mT (3.4 mT) and a *DP* of 0.44 ± 0.09. Skewness for the HCC, ICC, LCC, is 0.86 ± 0.04, 0.089 ± 0.04, and 1.04 ± 0.14 respectively (note that skewness of 1 is equivalent to a normal distribution^36^). The HCC is interpreted to represent a detrital magnetite phase inherited from parent materials (note the increased contribution to remanence of the HCC in typical background samples; Fig. 4c). The LCC is likely a low-coercivity MD magnetite phase, or a possible low-field tail for the ICC representing an artifact of component fitting. The origin of the ICC is interpreted to be pedogenic and is discussed in more detail below.

**Figure 5:**
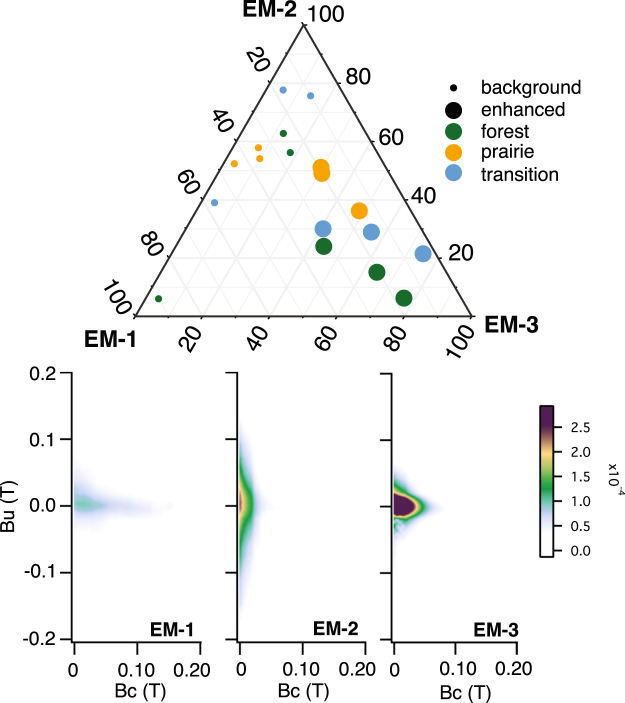
Ternary diagram and end member first-order reversal curve diagram results from FORCem analysis^37^. EM-1 is interpreted to represent detrial single domain magnetite inherited parent material. Coarser, detrital magnetite in the multi-domain state is represented by EM-2. Pedogenic magnetite, a mixture of SP and SSD magnetite is represented by EM-3. All enhnaced specimen are enriched in pedogenic EM-3, however prairie soils have higher contributions from detrital EM-1 and EM-2. Examples of individual FORC diagrams are provided in Figs S6–S8. The color scale is consistent for all FORC diagrams. For FORCem score plot, see Fig. S9.

**Figure 6:**
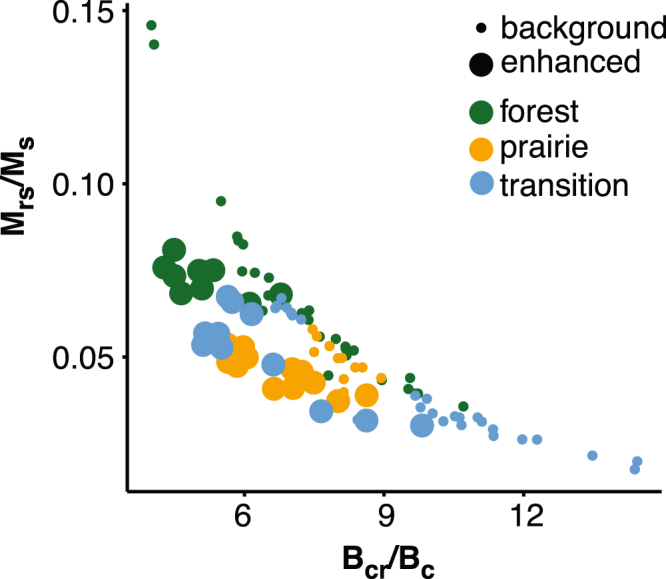
Day Plot of magnetic hysteresis properties. Background and enhanced specimen determined by criteria highlighted in Fig. S5. Clear trends can be observed for background and enhanced specimen from all study sites and strong differentiation is observed between the enhanced forest and prairie data.

First-order reversal curves for background and enhanced specimens from each profile record contributions from three distinct end members (Fig. 5). The strong isolated contributions along the central ridge along with vertical spread at low coercivity observed in the first end member (EM-1) is diagnostic of PSD magnetite^37,38^. The clear spread about the vertical axis observed in the second end member (EM-2) is characteristic of MD magnetite^38^ (Fig. 5). The observed FORC distribution for the third end member (EM-3) is consistent with a mixture of interacting SP and SSD grains of magnetite^38^ (Fig. 5). Contributions of EM-1 and EM-2 to overall magnetization are variable, but mostly consistent between background specimens (Fig. 5). There is a clear distinction between enhanced and background specimens driven by an increase in the contribution of EM-3. Enhanced forest and transitional specimens are generally more enriched in EM-3 comparative to enhanced prairie specimens (Fig. 5). We interpret EM-1 and EM-2 to represent detrital magnetite that is inherited from the parent material (glacial till and loess), while EM-3 is interpreted as pedogenic magnetite. Forest and prairie soils can be differentiated in a plot of squareness (*B*_*cr*_/*B*_*c*_) versus remanence ratio (*M*_*r*_/*M*_*s*_), where prairie soils show generally lower *M*_*r*_/*M*_*s*_ values, consistent with a relative enrichment in the fraction of coarse grained MD magnetite, super fine-grained SP magnetite, or both (Fig. 6).

Temperature dependent experiments (described in Methods) indicate that magnetite, and partially oxidized magnetite are the dominant magnetic mineral for all studied samples (see Supplementary Figs S10 and S11). Contributions of so-called ‘antiferromagnetic’ minerals such as goethite and hematite are minimal. However, goethite is identified by increased magnetization with cooling during thermal cycling^39^ and appears to to be present in enhanced forest specimens (Fig. S10). In addition, iron concretions were observed during sampling in the parent materials of forest soils. Soft iron masses are common in Des Moines Lobe till, so it is possible these concretions are either inherited from the parent material or formed in place. We also note that the presence of goethite in forest soils may be important to distinguishing variable soil processes across this soil transect, as discussed in more detail below.

## Discussion and Conclusions

This work represents, to our knowledge, the most rigorous evaluation of the effects of changing vegetation on magnetic mineral production in soils, in particular for soils that developed under uniform climate. The observed consistency in relative *χ*_*fd*_ and *χ*_*ARM*_/*IRM* in topsoils across the study transect suggests that biomediated redox processes^8^ leading to the production of SP/SSD magnetites in soils occur despite the influence of variable soil type and vegetation. Further, the median coercivity and dispersion reported for the ICC from coercivity analyses agrees well with previous studies that have isolated pedogenic magnetites from soils ranging across the globe^6,11,40^ and we interpret the ICC reported here to be pedogenic magnetite. Pedogenic magnetite contributes ∼45% of *M*_*r*_ for enhanced forest and prairie specimens and shows a decreasing pattern with depth similar to trends observed in *χ*_*fd*_ and *χ*_*ARM*_/*IRM* (Fig. 4a). There is also clear consistency in EM-3 from FORCem analyses (Fig. 5) that supports the hypothesis that pedogenic SP/SSD magnetite consistently dominates the magnetization of enhanced soil horizons across the transect. Together, our data set supports a pedogenic population of magnetite that is formed in soils independent of changing vegetation that is generally comparable to pedogenic magnetite populations recovered in soils developing under variable conditions throughout the world.

Climatic interpretations based on the magnetic mineralogy of paleosols are based on the fundamental assumption that ambient climate conditions are the principal control on the production of fine-grained, SP/SSD magnetite in upper soil horizons. To test the predictive power of recent paleoprecipitation proxies calibrated using pedogenic magnetite in loessic soils of the Great Plains^6^ we reconstructed precipitation for each sampling zone based on the *χ*_*ARM*_/*IRM* of the enhanced horizons (Fig. S12). Estimates for mean annual precipitation (MAP) are within ∼8% of the observed value (Fig. S12) and have good agreement across sampling zones. We again note that the *χ*_*ARM*_/*IRM* ratio is a relative indicator for the contribution of SSD grains to total remanence- and so this magnetic property only captures pedogenic magnetite that falls into the SSD grain size range. Accordingly, these results suggest that pedogenic production of SSD magnetite in soils is consistent enough with respect to changing vegetation that climatic inferences can still be made based on magnetic mineral assemblages.

Yet, increased *χ* and induced magnetization in prairie topsoils indicate an overall increase in the concentration of magnetic material in prairie soils compared to forest and transitional soils, which complicates climatic interpretations. For example, pedogenic susceptibility (*χ*_*ped*_, equivalent to the ${\chi }_{enhanced}-{\chi }_{background}$), which is used as a climatic indicator^1,2^, is much greater in prairie soils (57.5 ± 26.5 × 10^−8^ m^3^kg^−1^ compared to forest and transitional soils (8.43 ± 10.2 ×10^−8^ and 11.5 ± 27.9 × 10^−8^ m^3^kg^−1^, respectively) and overall variability for *χ*_*ped*_ between individual profiles is extremely high. Increased absolute *χ*_*fd*_ suggests an increased concentration of SP magnetite in prairie topsoils (Figs 2, 3), which contributes to the large differences observed between *χ*_*fd*_ of prairie soils compared with forest. In addition, end member contributions from FORCem unmixing (Fig. 5) on a subset of specimen suggest that the increase in concentration of magnetic material in prairie soils is due to a relative enrichment in prairie soils of detrital MD magnetite (EM-1, see Fig. 5). Together, these data suggest an increase in the concentration of SP magnetite and a relative enrichment in detrital MD magnetite in prairie soils. These differences suggest variable soil processes act across the study transect and complicate climatic interpretations made from parameters for the concentration grain-size fractions like absolute *χ*_*fd*_ and *ARM*.

It is important to constrain the soil processes that lead to the relative enrichment of magnetite in prairie soils, which may be the result of either a loss of magnetite from forest soils or additional inputs into prairie soils. One possible pathway for the production of magnetite in prairie soils may be burning, which is known to produce fine grained magnetite in top soil and is a key factor in determining the boundary of the forest-to-prairie transition in this region. However, if burning is the primary process responsible for the increased induced magnetization in prairie soils, then we would expect the elevated magnetization to be restricted to the uppermost 5–10 cm of soil. Yet, we observe elevated induced magnetizations down to 40 cm depth in prairie soils (Fig. 2). It is possible that plowing of prairie soils in the 1950s may be partially responsible for homogenizing the top soil, although plows used at this time usually only affected the top ∼20 cm of soils and it is unclear from aerial photos if plowing affected this locality at all (see Fig. S3). Finally, although early work highlighted burning as a process that can produce magnetites in top soils, more recent work has shown that it is not likely to be a primary driver for magnetic enhancement^41^.

Increased PET in prairie soils is related to changes in vegetation from forest to prairie and also increased wind speeds on the prairie, likely due to decreased tree cover to act as wind blocks. Given the increased PET and associated decrease in W from forest to prairie soils, we suggest that overall production of SP/SSD pedogenic magnetite is enhanced in prairie soils. This is consistent with the clear differences in the absolute concentration of SP magnetite shown from plots of absolute *χ*_*fd*_ with depth (Fig. 2b). Possible increases in detrital MD magnetite in prairie soils (indicated from FORCem analysis) may be related to eolian deposition of magnetic minerals on the prairie similarly related to increased wind speeds. However, an additional consequence of increased W in forest and transitional soils is the possibility for dissolution of magnetite during episodes of prolonged soil saturation. Dissolution of magnetite in forest sites is further facilitated by increasingly acidic soil conditions^42,43^. Measurements of pH on soil horizons from this study are in agreement with previous work^31^ and show that forest soil, particularly top soil, is more acidic compared to prairie and transitional soils (Table 1). Goethite is favored in soils with lower pH^42,43^ and is detectable only in enhanced forest top soil where conditions are most acidic (forest A horizons pH = 6.85). The cross plot of absolute *χ*_*fd*_ and *ARM* (Fig. 3a) highlights that forest soils (particularly enhanced samples) display a trend consistent with increased dissolution of SP magnetite relative to prairie and transitional soils. In contrast, given the similar trends displayed by transitional and prairie soils - it appears that transitional soils simply produce less SP and SSD magnetite than prairie soils. It follows that the progressive increase in magnetite observed in prairie soils relative to forest and transitional soils (Fig. 5) is likely related to some combination of these processes, all of which have no direct relationship with changes in climate.

**Table 1:** Average pH for soil horizons across study transect. Standard deviations are reported in parentheticals. All horizons match those displayed in Fig. 1.

Horizon	Prairie	Transitional	Forest
A	7.74 (0.25)	7.85 (0.23)	6.85 (0.31)
A2	—	8.07 (0.05)	—
Bw	7.87 (0.16)	—	—
E	—	—	7.03 (0.27)
Bt	—	7.94 (0.22)	7.03 (0.27)
BtC	—	—	7.15 (0.20)
C	8.01 (0.22)	7.67 (0.39)	—

An equilibrium balance between magnetic mineral formation and dissolution with respect to soil conditions is essential for a stable population of magnetic minerals to develop in soils in response to long term climatic and environmental conditions. Here, we present evidence in support of three important conclusions regarding mixed assemblages of magnetic minerals in soils. First, changes in soil moisture can be unrelated to changing climate and are likely to affect climatic interpretations based on pedogenic magnetic mineral assemblages - particularly for magnetic properties that isolate the concentration of SP or SSD magnetite (e.g., absolute *χ*_*fd*_ or *ARM*). Second, contributions from detrital magnetic minerals to the overall magnetization of soil samples complicate signals from pedogenic minerals. Detrital magnetic minerals are by definition not formed in soil, are subject to long-term dissolution processes, and are very unlikely to be in equilibrium with ambient climatic conditions. As a result, climatic interpretation based on bulk magnetic properties of soils without the removal of detrital signals is likely to be poorly constrained and uncertain. Finally, the SP/SSD population of magnetite in enhanced soil horizons across the study transect is highly consistent in grains-size and relative contribution to magnetization, is easily identifiable using a range of targeted magnetic parameters and techniques, and suggests that the processes controlling magnetite pedogenesis occurs independent of vegetation cover. Yet, dissolution processes diminish magnetic minerals including pedogenically produced magnetite. Based on the consistency reported here for relative measures of pedogenic magnetite across the transect, it is apparent that equilibrium conditions are reached between formation and dissolution processes with respect to the ambient climatic conditions. Thus, paleoclimate proxies that are based on ratios between the magnetic properties of enhanced and parent horizons may be more robust than those that are based on absolute magnetization values.

## Methods

Soil samples were collected from a combination of freshly dug soil pits, slide-hammer cores, and augered samples. Augered samples were collected from the inside of soil clods to avoid contamination. Soil color for wet samples was recorded using a Munsell color chart. All samples were dried, lightly crushed to homogenize, and sieved to remove soil particles larger than 5 mm. Specimens for magnetic measurements were prepared by packing soil samples into diamagnetic plastic cubes and securing with a non-magnetic potassium silicate adhesive. Magnetic measurements were conducted at the Institute for Rock Magnetism at the University of Minnesota. All specimens in this study (n = 98) were evaluated for magnetic susceptibility (*χ*, m^3^kg^−1^), frequency dependence of susceptibility (*χ*_*fd*_, % or m^3^kg^−1^, see below), isothermal remanent magnetization (*IRM*, Am^2^kg^−1^), anhysteretic remanent magnetization (*ARM*, Am^2^kg^−1^), and hysteresis properties. Magnetic susceptibility was measured at low (465 Hz, low frequency susceptibility is reported as *χ*) and high (4650 Hz) frequencies using a Magnon variable frequency susceptibility meter in an alternating current (AC) field of 300 Am^−1^. Relative *χ*_*fd*_ was calculated as a percentage, where *χ*_*fd*_ = (${\chi }_{465Hz}-{\chi }_{4650Hz})/{\chi }_{465Hz}\times 100$. Absolute *χ*_*fd*_ was calculated as the difference between ${\chi }_{465Hz}-{\chi }_{4650Hz}$ and is reported in units of m^3^kg^−1^. *IRM* was imparted using three pulses of a 100 mT direct current (DC) field in a pulse magnetizer and *ARM* was imparted in a peak alternating field (AF) of 100 mT in the presence of a weak DC bias field of 50 *μ*T. Both *IRM* and *ARM* were measured using a 2G Enterprises 760-R SQUID magnetometer within a shielded room with a background field of less than 100 nT. The susceptibility of *ARM* (*χ*_*ARM*_, mA^−1^) is calculated by dividing *ARM* by the bias field. Enhanced and background samples were determined by threshold criteria for *χ*_*fd*_ and *χ*_*ARM*_/*IRM*, where specimen with *χ*_*fd*_ > 2% and *χ*_*ARM*_/*IRM* > 4.5 × 10^−4^ mA^−1^ were categorized as enhanced and all other specimen were determined to be background.

Hysteresis loops and backfield remanence curves were measured using a Princeton Measurements Corporation Micromag vibrating sample magnetometer (VSM) at room temperature in fields up to 1 T. Saturation magnetization (*M*_*s*_, Am^2^kg^−1^) and coercivity (*B*_*c*_, mT) are determined from hysteresis loops, while saturation remanent magnetization (*M*_*rs*_, Am^2^kg^−1^) and coercivity of remanence (*B*_*cr*_, mT) are calculated from backfield curves^35^. Coercivity spectra were derived for all specimens as the absolute value of the first derivative of backfield curves. Coercivity unmixing was performed using MAX UnMix^36^, a new program for coercivity unmixing based on previous work^44–46^ (available online at www.irm.umn.edu/maxunmix).

A subset of samples, both background and enhanced, were analyzed using more sophisticated measurements in order to better constrain grain size distributions and magnetic mineralogy. An initial room temperature (300 K) remanence (RT-S*IRM*) imparted using a 5 T DC field (followed by 2.5 T pulse along same axis to minimize recoil within system) was measured during cooling to 20 K and warming back to room temperature using a Quantum Design Magnetic Properties Measurement System (MPMS). Field cooled (FC) and zero-field cooled (ZFC) remanence (2.5 T) was measured on cooling from 300 K to 20 K. RT-S*IRM* and FC-ZFC curves reveal remanence loss at diagnostic transitions, for example the Verwey transition for magnetite. To characterize magnetic grain size distributions, first order reversal curve (FORC) diagrams were measured using a Micromag-VSM. All FORC diagrams were processes using FORCinel v3.0 and smoothed using the simple smooth functionality with a smoothing factor of 5^47^. Decomposition of FORC diagrams was performed using FORCem^37^. FORCem unmixes FORC data using a principle component approach that allows for quantification of end member contributions to magnetization.

### Data availability

All data generated or analysed during this study are included in this published article (and its Supplementary Information files).

## Electronic supplementary material

supplemental information

supplemental dataset

## References

[1] Maher B, Thompson R, Zhou L (1994). Spatial and temporal reconstructions of changes in the Asian palaeomonsoon: A new mineral magnetic approach. Earth and Planetary Science Letters.

[2] Maher BA, Thompson R (1995). Paleorainfall reconstructions from pedogenic magnetic susceptibility variations in the Chinese Loess and Paleosols. Quaternary International.

[3] Maher BA (1998). Magnetic properties of modern soils and Quaternary loessic paleosols: paleoclimatic implications. Palaeogeography, Palaeoclimatology, Palaeoecology.

[4] Maher BA (2007). Environmental magnetism and climate change. Contemporary Physics.

[5] Geiss CE, Zanner CW (2007). Sediment magnetic signature of climate in modern loessic soils from the Great Plains. Quaternary International.

[6] Geiss CE, Egli R, Zanner CW (2008). Direct estimates of pedogenic magnetite as a tool to reconstruct past climates from buried soils. Journal of Geophysical Research.

[7] Balsam WL (2011). Magnetic susceptibility as a proxy for rainfall: Worldwide data from tropical and temperate climate. Quaternary Science Reviews.

[8] Orgeira, M. J., Egli, R. & Compagnucci, R. H. A quantitative model of magnetic enhancement in loessic soils. In Petrovský, E., Ivers, D., Harinarayana, T. & Herrero-Bervera, E. (eds) *The Earth’s Magnetic Interior*, 361–397 (Springer, Dordrecht, Netherlands, 2011).

[9] Long X, Ji J, Balsam W (2011). Rainfall-dependent transformations of iron oxides in a tropical saprolite transect of Hainan Island, South China: spectral and magnetic measurements. Journal of Geophysical Research.

[10] Liu, Q. *et al*. Environmental magnetism: principles and applications. *Review of Geophysics* **50**, RG4002 (2012).

[11] Maxbauer DP, Feinberg JM, Fox DL (2016). Magnetic mineral assemblages in soils and paleosols as the basis for paleoprecipitation proxies: a review of magnetic methods and challenges. Earth-Science Reviews.

[12] Lovley DR, Stolz JF, Nord GL, Phillips EJ (1987). Anaerobic production of magnetite by a dissimilatory iron-reducing microorganism. Nature.

[13] van Velzen AJ, Dekkers MJ (1999). Low-temperature oxidation of magnetite in loess-paleosol sequences: a correction of rock magnetic parameters. Studia Geophysica et Geodaetica.

[14] Chen T (2010). Characteristics and formation mechanism of pedogenic hematite in Quaternary Chinese loess and paleosols. Catena.

[15] Jenny, H. *Factors of Soil Formation* (McGraw-Hill, New York, 1941).

[16] Hatfield RG (2014). Particle size-specific magnetic measurements as a tool for enhancing our understanding of the bulk magnetic properties of sediments. Minerals.

[17] Heslop D (2015). Numerical strategies for magnetic mineral unmixing. Earth-Science Reviews.

[18] Porter SC, Hallet B, Wu X, An Z (2001). Dependence of Near-Surface Magnetic Susceptibility on Dust Accumulation Rate and Precipitation on the Chinese Loess Plateau. Quaternary Research.

[19] Hyland E, Sheldon ND, Van der Voo R, Badgley C, Abrajevitch A (2015). A new paleoprecipitation proxy based on soil magnetic properties: implications for expanding paleoclimate reconstructions. Geology.

[20] Maher B, Possolo A (2013). Statistical models for use of palaeosol magnetic properties as proxies of palaeorainfall. Global and Planetary Change.

[21] Heslop D, Roberts AP (2013). Calculating uncertainties on predictions of palaeoprecipitation from the magnetic properties of soils. Global and Planetary Change.

[22] Stinchcomb, G. E. & Peppe, D. J. The influence of time on the magnetic properties of late Quaternary periglacial and alluvial surface and buried soils along the Delaware River, USA. *Frontiers in Earth Science* (2014).

[23] Maher BA, Hu M (2006). A high-resolution record of Holocene rainfall variations from the western Chinese Loess Plateau: antiphase behaviour of the African/Indian and East Asian summer monsoons. The Holocene.

[24] Vidic NJ, Singer MJ, Verosub KL (2004). Duration dependence of magnetic susceptibility enhancement in the Chinese loess–palaeosols of the past 620 ky. Palaeogeography, Palaeoclimatology, Palaeoecology.

[25] Fine P, Singer MJ, Ven RLA, Verosub K, Southard RJ (1989). Role of Pedogenesis in Distribution of Magnetic Susceptibility in Two California Chronosequences. Geoderma.

[26] Hanesch M, Scholger R (2005). The influence of soil type on the magnetic susceptibility measured throughout soil profiles. Geophysical Journal International.

[27] Blundell A, Dearing J, Boyle J, Hannam J (2009). Controlling factors for the spatial variability of soil magnetic susceptibility across England and Wales. Earth-Science Reviews.

[28] Boyle JF, Dearing JA, Blundell A, Hannam JA (2010). Testing competing hypotheses for soil magnetic susceptibility using a new chemical kinetic model. Geology.

[29] de Jong E, Nestor P, Pennock D (1998). The use of magnetic susceptibility to measure long-term soil redistribution. Catena.

[30] de Jong E, Pennock D, Nestor P (2000). Magnetic susceptibility of soils in different slope positions in Saskatchewan, Canada. Catena.

[31] Severson RC, Arneman HF (1973). Soil characteristics of the forest-prairie ecotone in northwestern minnesota. Soil Science Society of America Journal.

[32] Lusardi BA, Jennings CE, Harris KL (2011). Provenance of des moines lobe till records ice-stream catchment evolution during laurentide deglaciation. Boreas.

[33] Clark JS (1990). Fire and climate change during the last 750 years in northwestern minnesota. Ecological Monographs.

[34] Dearing JA (1996). Frequency-dependent susceptibility measurements of environmental materials. Geophysical Journal International.

[35] Tauxe, L., Banerjee, S. K., Butler, R. & van der Voo, R. *Essentials of Paleomagnetism* (University of California Press, 2014), 3rd web edn.

[36] Maxbauer DP, Feinberg JM, Fox DL (2016). Max unmix: A web application for unmixing magnetic coercivity distributions. Computers & Geosciences.

[37] Lascu I (2015). Magnetic unmixing of first-order reversal curve diagrams using principle component analysis. Geochemistry, Geophysics, and Geosystems.

[38] Roberts AP, Heslop D, Zhao X, Pike CR (2014). Understanding fine magnetic particle systems through use of first-order reversal curve diagrams. Reviews of Geophysics.

[39] Maher BA, Karloukovski VV, Mutch TJ (2004). High-field remanence properties of synthetic and natural submicrometre haematites and goethites: significance for environmental contexts. Earth and Planetary Science Letters.

[40] Egli R (2004). Characterization of individual rock magnetic components by analysis of remanence curves 1. Unmixing natural sediments. Studia Geophysica et Geodaetica.

[41] Quinton EE, Dahms DE, Geiss CE (2011). Magnetic analyses of soils from the Wind River Range, Wyoming, constrain rates and pathways of magnetic enhancement for soils from semiarid climates. Geochemistry, Geophysics, Geosystems.

[42] Cornell, R. & Schwertmann, U. *The Iron Oxides: Structures, Properties, Reactions, Occurences, and Uses* (Wiley-VCH, 2003), 2nd edn.

[43] Maher B, Alekseev A, Alekseeva T (2003). Magnetic mineralogy of soils across the Russian Steppe: climatic dependence of pedogenic magnetite formation. Palaeogeography, Palaeoclimatology, Palaeoecology.

[44] Kruiver PP, Dekkers MJ, Heslop D (2001). Quantification of magnetic coercivity components by the analysis of acquisition curves of isothermal remanent magnetisation. Earth and Planetary Science Letters.

[45] Heslop D, McIntosh G, Dekkers MJ (2004). Using time- and temperature-dependent Preisach models to investigate the limitations of modelling isothermal remanent magnetization acquisition curves with cumulative log Gaussian functions. Geophysical Journal International.

[46] Egli R (2003). Analysis of the field dependence of remanent magnetization curves. Journal of Geophysical Research.

[47] Harrison RJ, Feinberg JM (2009). Forcinel: An improved algorithm for calculating first-order reversal curve distributions using locally weighted regression smoothing. Geochemistry, Geophysics, and Geosystems.

