# Supplementary material for: Response of pedogenic magnetite to changing vegetation in soils developed under uniform climate, topography, and parent material
Source: Sci Rep. 2017 Dec 14;7:17575. doi: 10.1038/s41598-017-17722-2 (PMC5730611; doi:10.1038/s41598-017-17722-2)
Supplement: Supplementary file 1 — supplemental information [file 41598_2017_17722_MOESM1_ESM.pdf]

RESPONSE OF PEDOGENIC MAGNETITE TO CHANGING VEGETATION IN SOILS DEVELOPED UNDER UNIFORM CLIMATE, TOPOGRAPHY, AND PARENT MATERIAL

DANIEL P. MAXBAUER, JOSHUA M. FEINBERG, DAVID L. FOX, EDWARD A. NATER

SUPPLEMENTAL INFORMATION

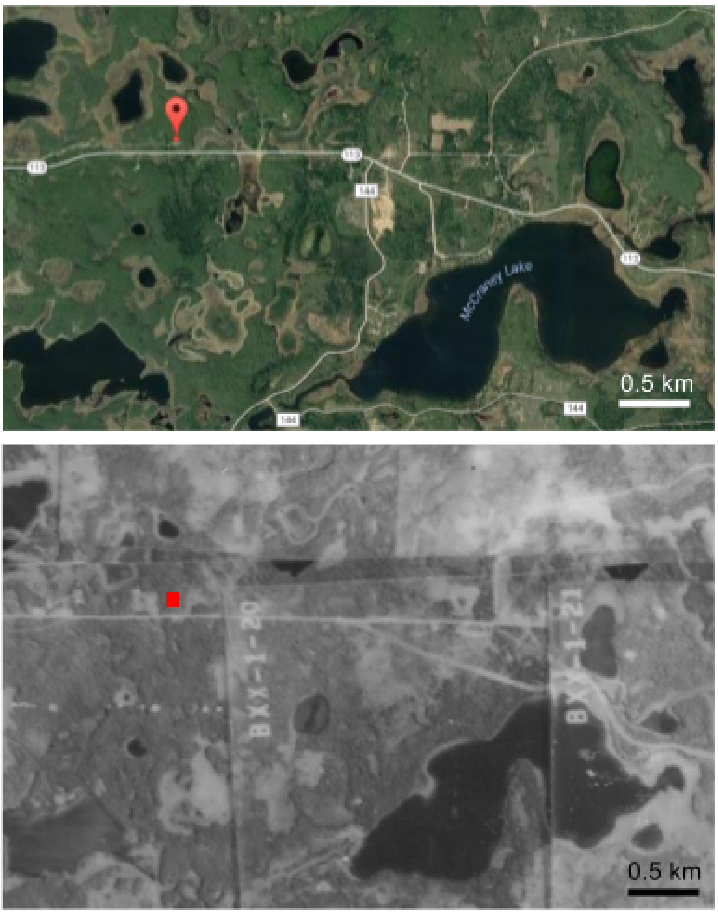

Figure 1. Aerial imagery comparing the sampling location for forest soils. Top image is from Google Earth (Map data: Google, DigitalGlobe) and can be compared to aerial photographs from 1939. Historical imagery is from the John R. Borchert Map Library, University of Minnesota using aerial photographs for Mahnomen County, Minnesota (available online here: http://geo.lib.umn.edu/aerial_photos/indexes/mahnomen.html). Sampling location in both images highlighted with a red marker. The Borchert library images for 1939 are found here: http://geo.lib.umn.edu/airphoto_indexes/Mahnomen39-01.jpg

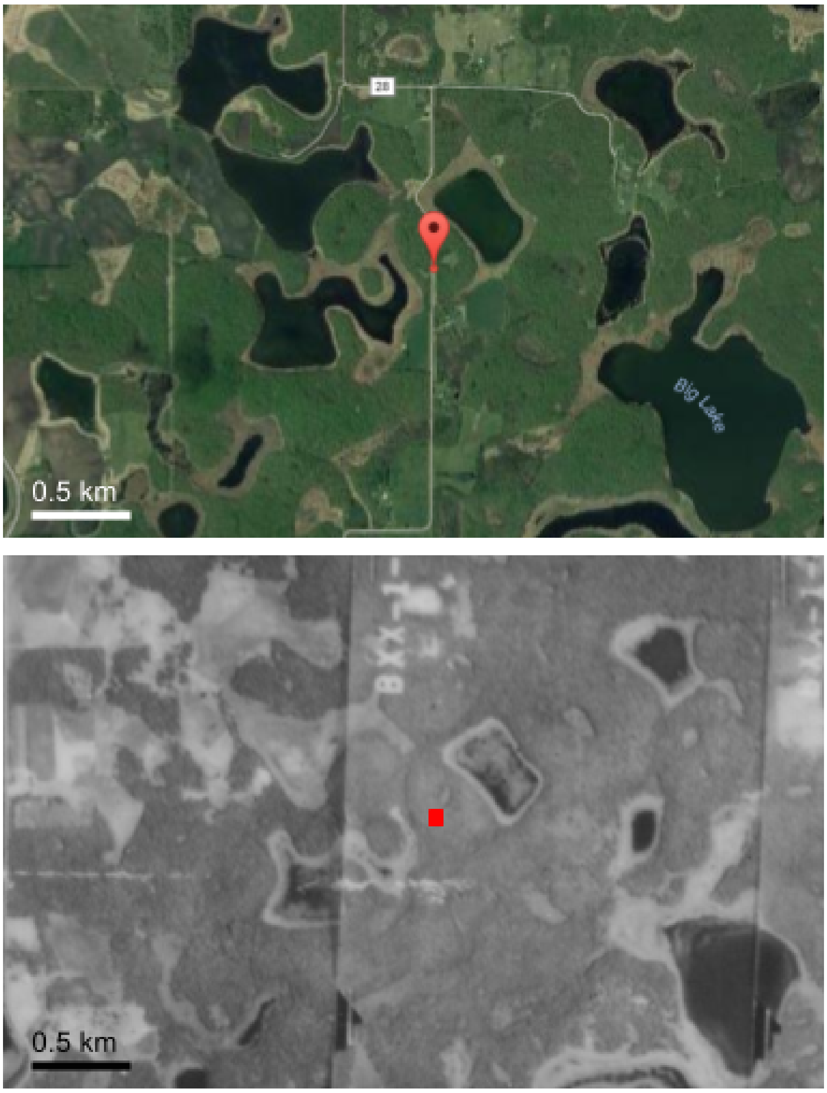

Figure 2. Aerial imagery comparing the sampling location for transitional soils. Top image is from Google Earth (Map data: Google, DigitalGlobe) and can be compared to aerial photographs from 1939. Historical imagery is from the John R. Borchert Map Library, University of Minnesota using aerial photographs for Mahnomen County, Minnesota (available online here: http://geo.lib.umn.edu/aerial_photos/indexes/mahnomen.html). Sampling location in both images highlighted with a red marker. The Borchert library images for 1939 are found here: http://geo.lib.umn.edu/airphoto_indexes/Mahnomen39-01.jpg

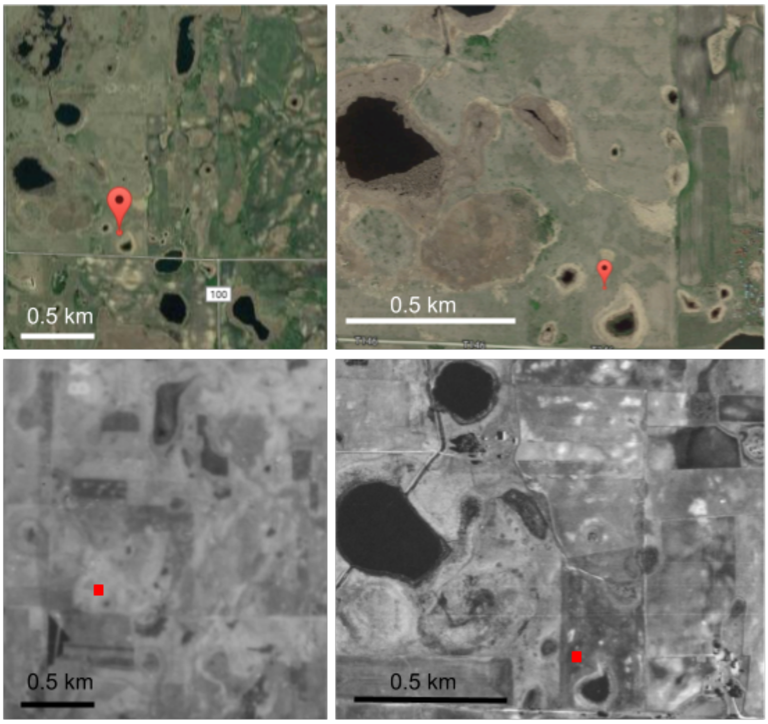

FIGURE 3. Aerial imagery comparing the sampling location for prairie soils. Top images are from Google Earth (Map data: Google, DigitalGlobe) and can be compared to aerial photographs from 1939 (bottom left) and 1953 (bottom right). There is apparent plowing in the 1953 image - and this likely effected mixing in the top ~20 cm of topsoil. Historical imagery is from the John R. Borchert Map Library, University of Minnesota using aerial photographs for Mahnomen County, Minnesota (available online here: http://geo.lib.umn.edu/aerial_photos/indexes/mahnomen.html). Sampling location in both images highlighted with a red marker. The Borchert library images for 1939 are found here:
http://geo.lib.umn.edu/airphoto_indexes/Mahnomen39-01.jpg
And for 1953 are found here:
http://geo.lib.umn.edu/airphoto_indexes/Mahnomen53-01.jpg

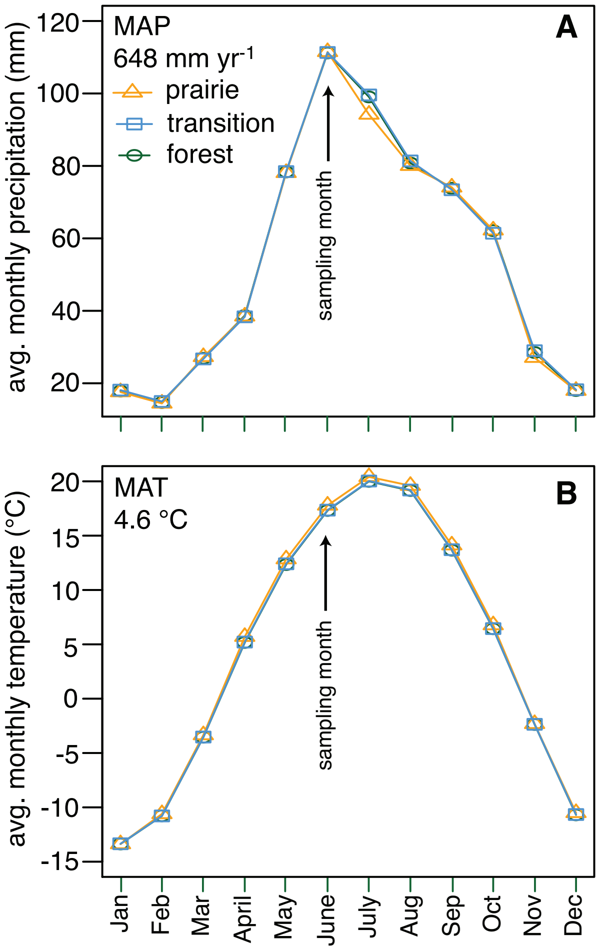

Figure 4. Average 30-year normal (1981-2010) monthly precipitation (**A**) and temperature (**B**) normals for each sampling locality. Annual precipitation and temperature averages are reported in the upper left of each panel. The sampling month (June) is indicated with arrows in each plot. Data from the PRISM Climate Group, Oregon State University, http://prism.oregonstate.edu, created 9 March 2016.

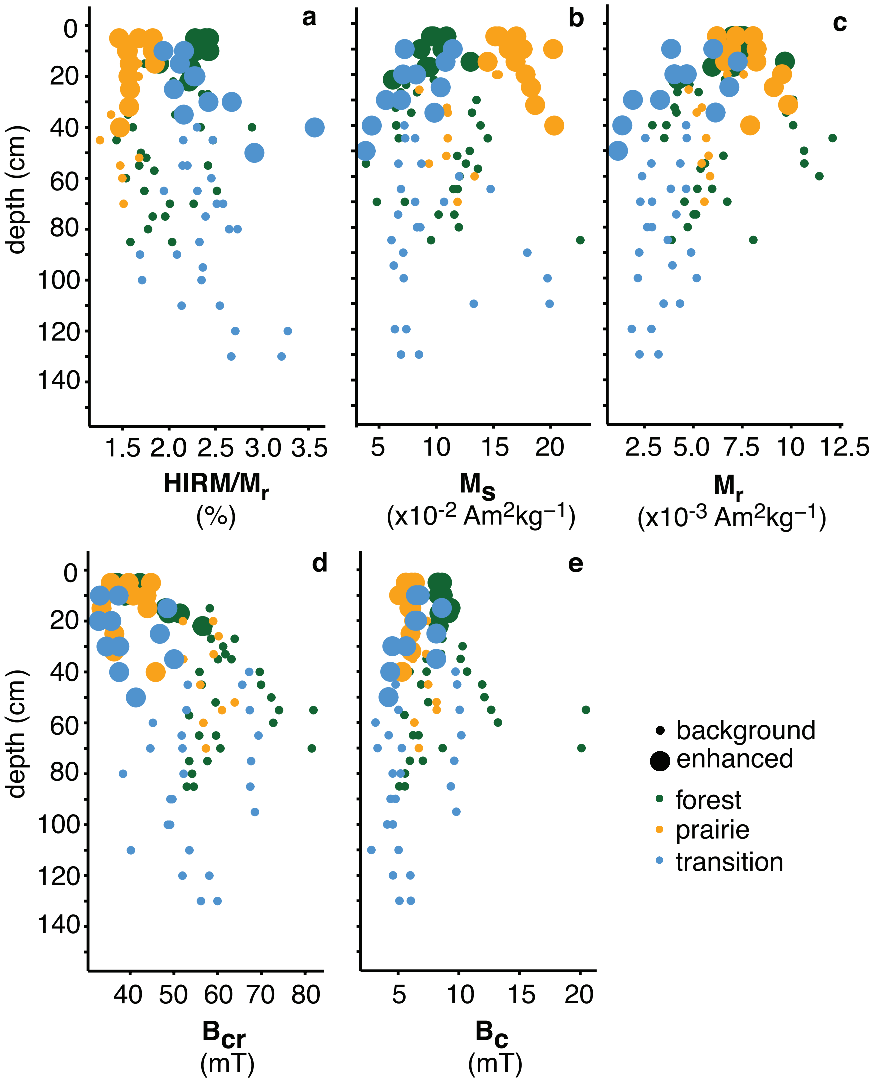

FIGURE 5. Magnetic properties with depth for soil transect. Data reported here for hard isothermal remanent magnetization ($HIRM$, calculated with 300 mT intermediate field) relative to saturation remanences (M_r) (a), saturation magnetization (M_s (b), saturation remanent magnetization (M_r) (c), coercivity of remanence (B_{cr} (d), and coercivity (B_c). Background and enhanced specimen in all cases are determined by criteria highlighted by shaded boxes in Figure 2d,e in the main body for this article.

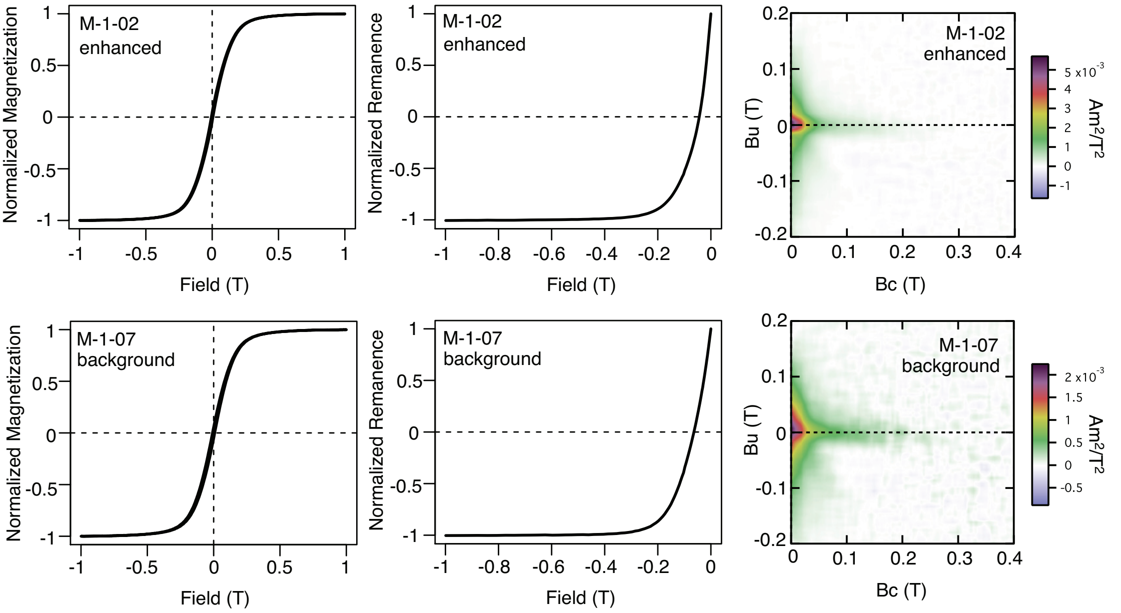

FIGURE 6. Hysteresis loops (left column), backfield remanence curves (middle column), and first order reversal curve (FORC) diagrams for representative enhanced and background prairie specimens M-1-02 and M-1-07. All FORC diagrams processed with a smoothing factor of 5 in FORCinel v3.0 using simple smooth (Harrison and Feinberg, 2008).

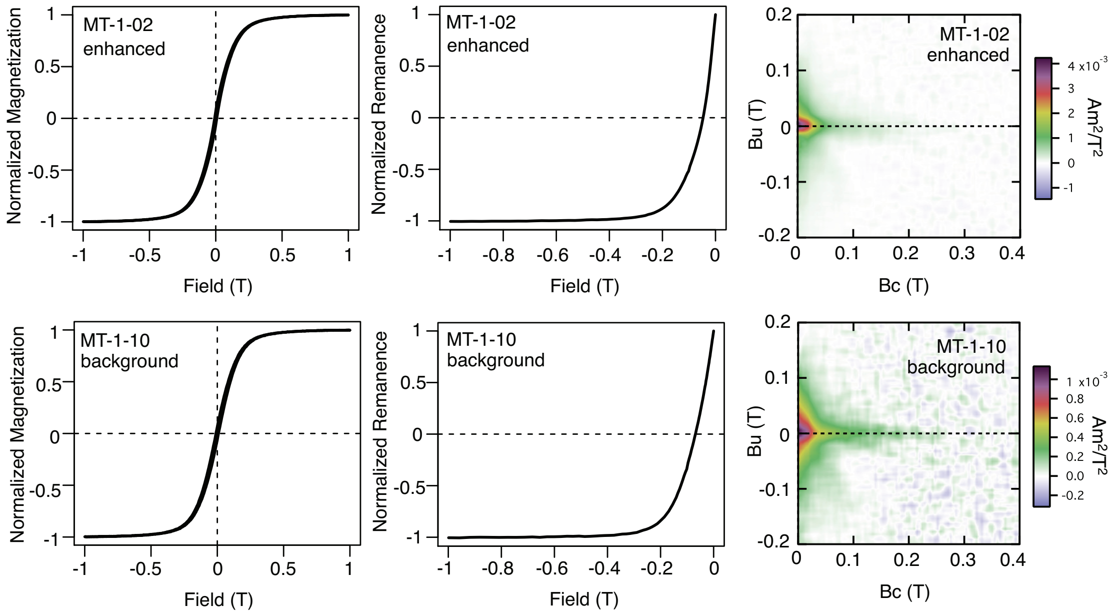

Figure 7. Hysteresis loops (left column), backfield remanence curves (middle column), and first order reversal curve (FORC) diagrams for representative enhanced and background transitional specimens MT-1-02 and MT-1-10. All FORC diagrams processed with a smoothing factor of 5 in FORCinel v3.0 using simple smooth (Harrison and Feinberg, 2008).

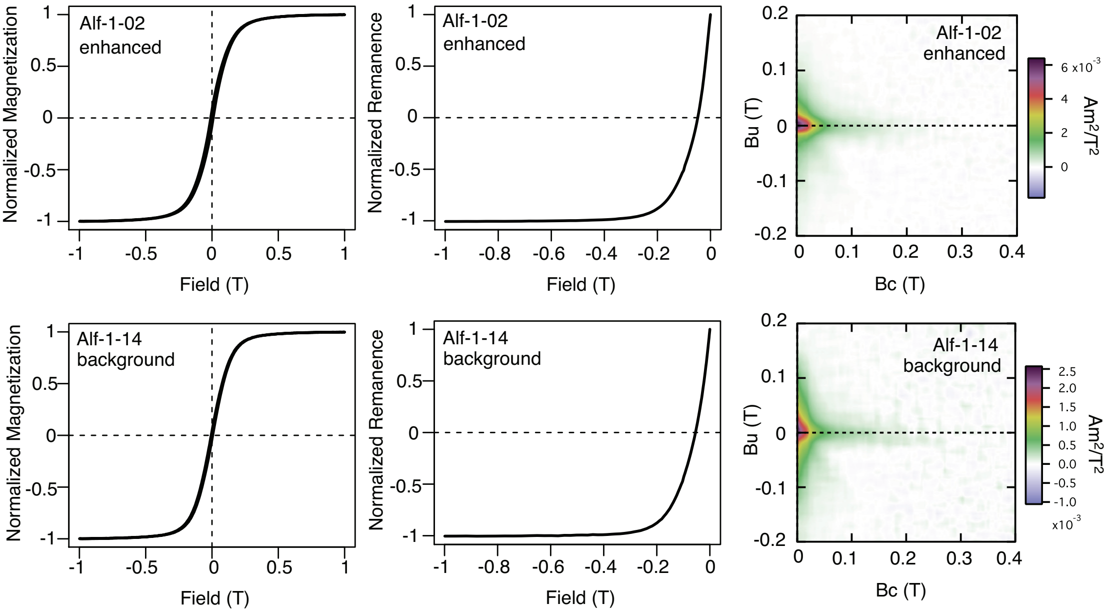

Figure 8. Hysteresis loops (left column), backfield remanence curves (middle column), and first order reversal curve (FORC) diagrams for representative enhanced and background forest specimens Alf-1-02 and Alf-1-14. All FORC diagrams processed with a smoothing factor of 5 in FORCinel v3.0 using simple smooth (Harrison and Feinberg, 2008).

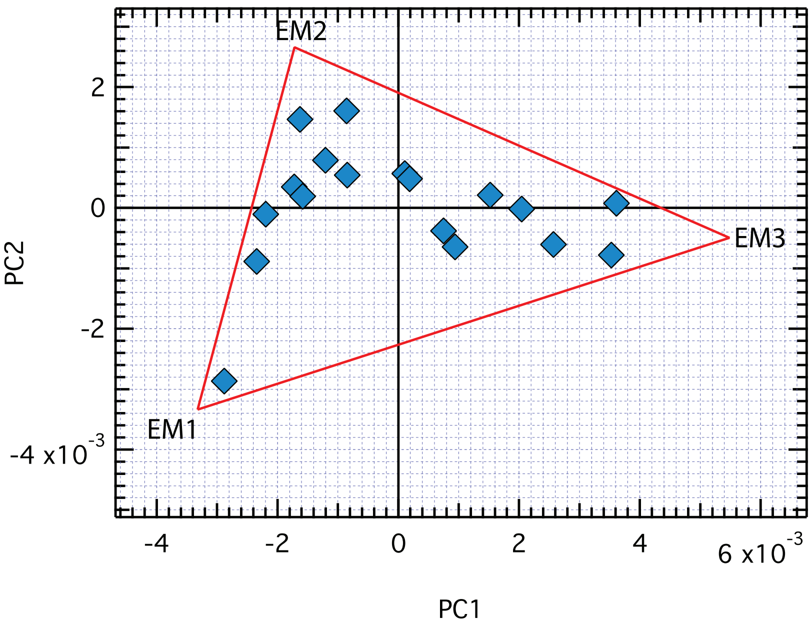

FIGURE 9. Score plot results from the PCA analysis within the FORCem analytical package. End member scores are represented by the corners of the triangle and all data falls within the mixing space delineated by this triangle. See Lascu et al. (2015) for details.

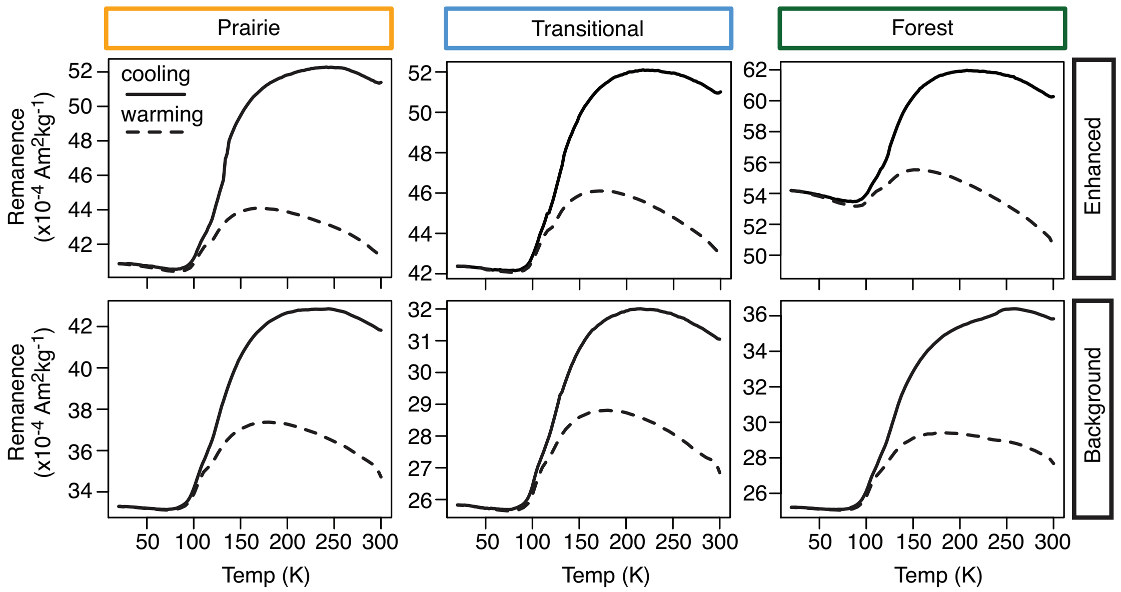

FIGURE 10. Room-temperature saturation isothermal remanent magnetization (RT-SIRM) curves for enhanced and background specimen from prairie, transitional, and forest sampling localities. All samples display a broad loss of Magnetization just prior to the Verwey transition of magnetite (110 K for pure, stoichiometric magnetite) indicating magnetic mineralogy dominated by magnetite and partially oxidized magnetite. The increase in magnetization with cooling observed in the forest enhanced specimen is indicative of contributions from goethite (Maher et al., 2004).

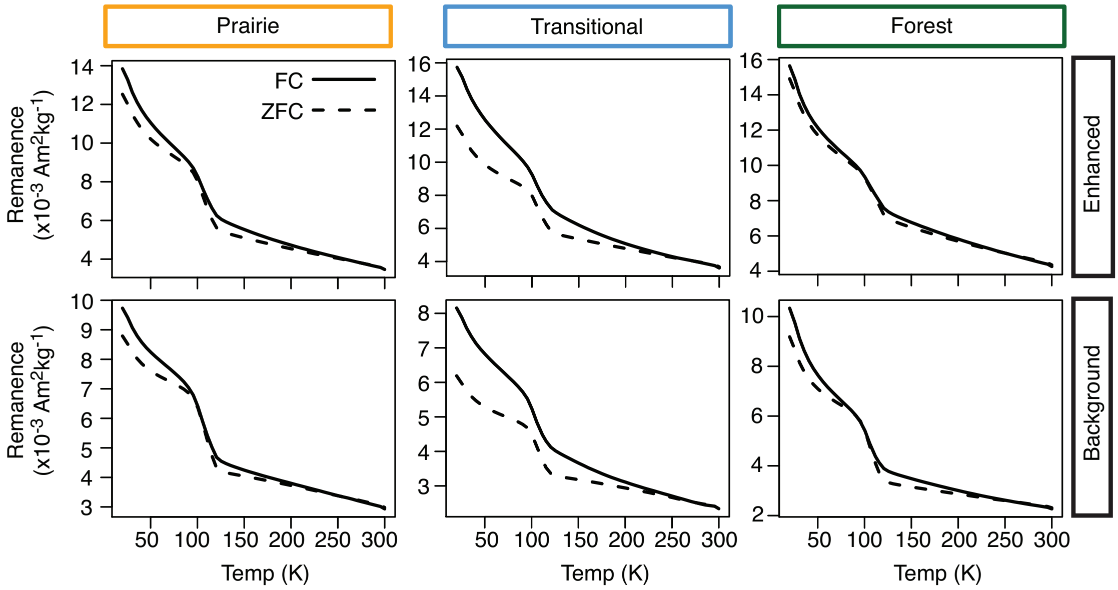

Figure 11. Field cooled (FC) and zero field cooled (ZFC) remanence (2.5 T) for enhanced and background specimen from each sampling locality. FC remanence that is greater than ZFC remanence is indicative of single domain magnetite dominating the remanence held by these specimens. The Verwey transition of magnetite is clearly observed in all specimen confirming that magnetite is the primary magnetic carrier in this system.

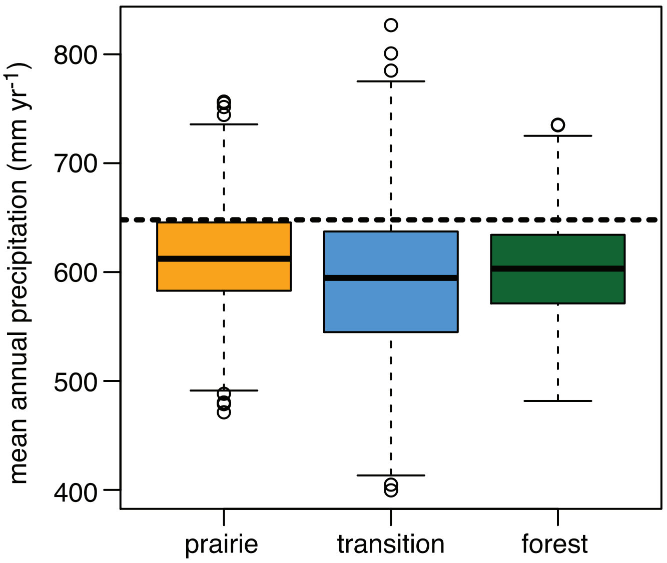

FIGURE 12. Estimates of mean annual precipitation (MAP) based on the *ARM*/*IRM* proxy of Geiss et al. (2008). For each sampling zone, *ARM*/*IRM* for enhanced specimens was resampled 10,000 times using mean and standard deviations, assuming normality, and MAP was reconstructed from each resampled value of *ARM*/*IRM*. Box plots display median (thick center bar) 50% (colored box) and 95% (dashed vertical lines) confidence limits. Outliers beyond 95% confidence are shown as open symbols. The thick horizontal dashed line highlights the observed 30 year normal precipitation (MAP = 648 mm yr^{-1} for the transect based on PRISM data; PRISM Climate Group, Oregon State University, http://prism.oregonstate.edu, created 9 March 2016).

References

Geiss, C.E., Egli, R., and Zanner, C.W., 2008. Direct estimates of pedogenic magnetite as a tool to reconstruct past climates from buried soils. *Journal of Geophysical Research.* 113, B11102.

Harrison,R.J., and Feinberg, J.M., 2008. FORCinel: An improved algorithm for calculating first-order reversal curve distributions using locally weighted regression smoothing, *Geochemistry, Geophysics, Geosystems.* 9, Q05016, doi:10.1029/2008GC001987.

Lascu, I., R. J. Harrison, Y. Li, J. R. Muraszko, J. E. T. Channell, A. M. Piotrowski, and D. A. Hodell (2015), Magnetic unmixing of first-order reversal curve diagrams using principle component analysis, *Geochemistry, Geophysics, and Geosystems.* 16, 2900?2915, doi:10.1002/2015GC005909.

Maher, B.A., Karloukovski, V.V., and Mutch, T.J., 2004. High-field remanence properties of synthetic and natural submicrometre haematites and goethites: significance for environmental contexts. *Earth and Planetary Science Letters.* 226, 491-505.
